# New Efforts to Demonstrate the Successful Use of TRH as a Therapeutic Agent

**DOI:** 10.3390/ijms241311047

**Published:** 2023-07-04

**Authors:** Elena Alvarez-Salas, Cinthia García-Luna, Patricia de Gortari

**Affiliations:** Laboratorio de Neurofisiología Molecular, Dirección de Neurociencias, Instituto Nacional de Psiquiatría Ramón de la Fuente Muñiz, Calzada México-Xochimilco 101, San Lorenzo Huipulco, Tlalpan, Mexico City CP 14370, Mexico; alvareze@imp.edu.mx (E.A.-S.); garcialuna@imp.edu.mx (C.G.-L.)

**Keywords:** thyrotropin-releasing hormone, therapeutic factor, neuromodulator, TRH analogs

## Abstract

Thyrotropin-releasing hormone (TRH) is a tripeptide that regulates the neuroendocrine thyroid axis. Moreover, its widespread brain distribution has indicated that it is a relevant neuromodulator of behaviors such as feeding, arousal, anxiety, and locomotion. Importantly, it is also a neurotrophic peptide, and thus may halt the development of neurodegenerative diseases and improve mood-related disorders. Its neuroprotective actions on those pathologies and behaviors have been limited due to its poor intestinal and blood–brain barrier permeability, and because it is rapidly degraded by a serum enzyme. As new strategies such as TRH intranasal delivery emerge, a renewed interest in the peptide has arisen. TRH analogs have proven to be safe in animals and humans, while not inducing alterations in thyroid hormones’ levels. In this review, we integrate research from different approaches, aiming to demonstrate the therapeutic effects of TRH, and to summarize new efforts to prolong and facilitate the peptide’s actions to improve symptoms and the progression of several pathologies.

## 1. Introduction

Thyrotropin-releasing hormone (TRH) was the first hypothalamic peptide to be purified and studied as a neuroendocrine factor [1,2]. When released from neurons of the hypothalamic paraventricular nucleus (PVN) to the portal blood, it stimulates the synthesis and release of thyrotropin (TSH), which reaches the thyroid gland and favors the synthesis and release of thyroid hormones into the bloodstream. Thus, metabolic rate, energy homeostasis, and thermogenesis are known to be under the control of TRH as a neurohormone [3].

In years following its purification, TRH’s neuromodulatory role was promptly unraveled and extensively evaluated, due to its role in releasing, activating, and counteracting some neurotransmitters’ actions, alongside modulating the levels of neurotrophic factors. TRH is known to act as a homeostatic factor, returning a specific hyperactive or inhibited neurotransmitter pathway to its basal functioning; this has been evidenced when both its analeptic and anticonvulsant effects have been observed [4,5].

However, many disadvantages have been found when trying to use TRH as a therapeutic agent. It is a hydrophilic molecule (pKa = 6.2); thus, it is impermeable to the blood–brain barrier (BBB). It is formed by three amino acids, pyroglutamyl–histidyl–prolinamide, and although both of its amine and carboxyl groups are modified to avoid degradation by non-specific peptidases, its cleavage is rapidly promoted by the highly specific serum enzyme thyroliberinase, which inactivates the peptide when intravenously (i.v.) administered; therefore, the duration of its effects is short (10 min in average), and it has potential undesired endocrine actions.

Different TRH analogs have been designed and tested in preclinical and clinical studies, and their specific characteristics have been reviewed elsewhere [6]. These analogs have distinct modifications in their peptide structure that make them more potent and stable than TRH alone; moreover, they are designed to be selective for the TRH-R2 receptor (mediating non-hypophysiotropic effects in rodents), thereby avoiding undesired TSH-releasing effects [7].

Even though the peptide’s use in therapy (and that of some of its analogs) was approved by the FDA, by the early 2000s, fewer and more sparse studies were being performed to support its therapeutic roles [8,9].

Several studies show that TRH administration may increase cardiac and respiratory rates or gut motility; it may be neuroprotective after trauma injuries or ischemia, and it is able to ameliorate cognitive dysfunctions in different neuropsychiatric illnesses such as Alzheimer’s disease (AD) and depression [6,9]. Moreover, even though some actions of TRH are long-known, such as its role in food intake regulation and the reversal of respiratory depression induced by narcotics, TRH is gaining more importance due to the current epidemics of obesity and fentanyl abuse. Interestingly, around ten years ago, new strategies for delivering the peptide arose, and new analogs were designed to overcome the pharmacological disadvantages of TRH [10].

Thus, we here aim to compile the therapeutic effects of TRH and its analogs on several diseases, symptoms (i.e., fatigue), and behaviors (i.e., food intake), where TRH’s neuromodulatory functions are more widely studied (Table 1). Furthermore, we have gathered and described some new approaches (intranasal delivery, continuous administration, use of BBB permeable analogs), performed in the last 5 years, that have helped researchers to reconsider the possibilities of TRH as a promising therapeutic factor in diverse central alterations.

### 1.1. TRH-Stimulated Breathing in Opioid-Induced Respiratory Depression

In recent years, one of the therapeutic actions of TRH that has been most tested is its ability to reverse the respiratory depression induced by opioids. Since the 1970s, many studies have shown that intracerebroventricular (i.c.v.) administration of TRH has counteracting effects on some actions of non-opioid drugs, such as the reversal of pentobarbital and ethanol-induced narcosis [28] and hypothermia [29], in rabbits and monkeys. Observations of an excitatory role of TRH in the neurons that regulate ventilation in animals, such as those from the dorsal respiratory group, area postrema, medulla oblongata, striatum, the phrenic motor nucleus, hypoglossal motoneurons, and in the preBötzinger complex [21], along with the high expression of the peptide and of its receptors in those areas, support the stimulatory effect of TRH on breathing when centrally administered.

When TRH and the analog L-pyro-2-aminoadipil-L-leucyl-L-proline amide (RGH 22012) are injected intravenously or onto the dorsal pontomedullary surface, they block the effects of opioids; these results showed the effectiveness of TRH in reversing the morphine-induced decreased respiratory rate in anesthetized rats [20,30]; additionally, TRH administration (100 nM) reversed breathing depression in brainstem–spinal cord preparations administered with morphine [31].

In humans, an intravenous injection of TRH (0.2, 0.4 mg) has also been proven to be effective in activating ventilation along with increased heart rate, in a dose-dependent manner, but only in the short term, and while provoking some uncomfortable sensations such as dizziness or agitation [32]. However, human studies are scarce, and have shown inconsistent results for supporting the administration of TRH as a breathing stimulatory therapy [33].

Given that respiratory depression is a frequent complication in patients with opioid treatment or abuse, TRH has again been evaluated as a possible therapeutic agent for reversing opioid-induced breathing depression; however, in humans, its effects have not been observed [21]. The peptide has been considered for use as an alternative to naloxone, an opioid antagonist that activates breathing, mainly because naloxone provokes unwanted side effects when administered to reverse depressed breathing caused by morphine or fentanyl treatments [21,34]. In addition, because in contrast to naloxone, TRH does not exert its effects through µ-opioid receptor (MOR) activation, an undesired decrease in analgesia (and narcosis produced by opioids) can be avoided.

In fact, since NMDA receptor antagonists such as MK-801 block TRH’s breathing stimulation in morphine-induced respiratory depression, NMDA has been proposed as a mediator of TRH’s effects, instead of acting through MOR [20,21,30]. Another advantage is that TRH has been approved by the FDA, which encouraged recent efforts to support its clinical use.

New studies in animals have confirmed the breathing stimulatory effect of a single intravenous injection of TRH (1 mg/kg), and of its analog taltirelin (1 mg/kg), by observing a higher ventilation rate in spontaneously breathing anesthetized rats (with isofluorane). These studies also evaluated a novel scheme for TRH delivery consisting of continuous intravenous administration of 1–5 mg/kg/h after the injection of a single bolus, which again supported that the effects of TRH in reversing the respiratory depressant action of morphine are dose-dependent [29].

Furthermore, the peptide and its analog when intravenously injected are able to reverse the effects of a lethal dose of morphine (21 ± 5 mg/kg in less than 5 min), and the actions of TRH are potentiated when simultaneously administered with naloxone, which supports the two different pathways of TRH and naloxone to block morphine-induced low ventilation rates [19].

Importantly, studies have also shown that TRH is not only able to reverse, but also to prevent the low ventilation rate caused by morphine when TRH is administered prior to the opioid [19]. Although TRH is ineffective in improving oxygenation, it can reduce the acidosis induced by morphine for a short term (15 min) [19], which is an unwanted side effect of opioid treatment.

By using an intratracheal delivery method, TRH and taltirelin administration has also been proven efficient in stimulating breathing, with a 40 min duration, when depressed respiration is induced by morphine. Some other observed effects are increased heart rate and blood pressure. However, as TRH and its analog do not change morphine-induced narcosis and analgesia, their advantage vs. naloxone use are supported by intratracheal delivery [19]. In awake rats, it has also been confirmed that an intravenous injection of taltirelin is able to reverse morphine and sufentanil-induced breathing depression [35], but also induces some undesirable effects, such as increasing morphine-induced lactic acidosis, probably due to the stimulation of breathing, or due to the provocation of muscle rigidity [34,35].

In humans, new studies supporting TRH and its analogs as a therapeutic route for reversing the breathing-depressing actions of remifentanil are not very promising, given that repeated intravenous infusions of TRH from 0.2 to 8 mg do not have any effect on patients’ ventilation rates [21]. There are not clear explanations for the discrepancies within the animals’ results; therefore, more efforts are needed to test new methods of delivery for TRH and its analogs, in order to improve its effects in patients under opioid treatment or suffering with opioid abuse.

### 1.2. Apnea/Respiratory Stimulation

The respiratory stimulant effects of TRH and its analogs are relevant for identifying them as potential therapeutic agents in breathing disorders other than opioid-induced respiratory depression, such as those presented by patients with obstructive sleep apnea (OSA).

Besides its approval by the FDA, the relevance for testing TRH and its analogs for ameliorating breathing disorder’s symptoms in OSA is the high expression of TRH receptors in the hypoglossal motor nucleus, the high content of afferent connections that this nucleus receives from TRH-synthesizing neurons, and the excitatory actions of the peptides in that nucleus. Furthermore, when intracerebroventricularly administered in rats, TRH is able to increase the rhythmic activity of the neurons in the nucleus of the solitary tract, thus contributing to enhancing the respiratory rate. Additionally, TRH and its analogs activate the norepinephrine and cholinergic pathways that impinge and stimulate the tongue and hypoglossal muscles through binding to the highly expressed α-adrenergic and M2 muscarinic receptors on those muscles; this, by consequence, modulates the upper airway respiratory system altered in OSA.

The TRH analog taltirelin, which has high blood–brain barrier (BBB) penetrating properties with no endocrine effects, has been microperfused into the hypoglossal motor nucleus and intraperitoneally (i.p.) administered to sleeping rats, showing the successful stimulating rates of the pharyngeal muscle and of the tongue motor activities of animals, in comparison with vehicle-injected groups. However, taltirelin also suppresses rapid-eye movement sleep (REM) and non-REM sleep in rats, and increases wakefulness, which is an undesirable effect for treating OSA patients.

Taltirelin’s effects are observed by the chronic electroencephalographic and electromyographic registers in the tongue, diaphragm, and neck muscles, which are activated when two doses (0.1 and 1.0 mg/kg) of the TRH analog are intraperitoneally administered to rats, with and without the addition of a drug able to increase the arousal threshold of animals, thus causing no alteration of their upper airway muscle activity (trazodone 30 mg/kg). Taltirelin is known to have high affinity for TRH receptors in the brain, in comparison to its low affinity for those of the pituitary gland, which is advantageous for avoiding endocrine effects, mainly if low doses of the analog are used. Additionally, taltirelin is resistant to the actions of a TRH-degrading enzyme present in serum, thyroliberinase.

Given the proven efficacy of TRH and its analogs in activating arousal and favoring awakening, as seen in the study of Liu et al. [24], its use will be more advantageous for increasing the respiratory rate in patients with drug-induced breathing depression when it is also important that narcosis be reversed; it will be less advantageous in obstructive sleep apnea, because awakening is an undesirable effect of treating these patients.

### 1.3. Hypothermia

By testing the proven ability of TRH to antagonize reserpine-induced hypothermia, the efficacy of different designed and synthesized analogs has been studied in vivo by oral and intravenous delivery. Kobayashi et al. [22] successfully obtained TRH stereoisomers with characteristics superior to those of the native peptide and some of its known analogs, for use in the clinic. They tested different analogs in mice with hypothermia using subcutaneous injections of reserpine, trying to mimic TRH’s central action in increasing the mice’s temperatures [22,23]. This central effect of TRH has previously been described, when administration of the peptide reversed ethanol-induced hypothermia in mice [36]. New approaches by Kobayashi and colleagues used oral and intravenous delivery routes, with promising results for some of the stereoisomers tested.

Reserpine’s hypothermic effects are due to the decreased activity of catecholaminergic and serotonergic neurons; these are depleted by the respective neurotransmitter (NT), as opposed to the proposed central effects of TRH as a neuromodulator, since the peptide enhances the synthesis and release of those NT in the CNS; thus, a reserpine-injected rodent model seems suitable for proving the anti-hypothermic actions of TRH mimetics.

The findings of Kobayashi et al. [22] support that the potency of reversing the reserpine hypothermic role of TRH may successfully be 100 times more potent when using some of the new tested TRH analogs in mice subcutaneously injected with reserpine that show a colonic temperature of 30 °C or less [22]. Another advantage of some TRH mimetics is that they do not have endocrine effects, which is relevant because hypothalamic PVN TRH directs the functioning of the thyroid axis and ultimately increases serum T_3_ and T_4_ levels. Thyroid hormones target the adipose tissue and stimulate the expression of thermic homeostasis-regulating enzymes that act on mitochondria and increase the temperature of the reserpine-injected mice, thereby confounding the specific actions of TRH analogs in the CNS. This characteristic has been observed in previously designed TRH mimetics, such as taltirelin, when orally administered in mice and in rabbits intraperitoneally injected with reserpine [37]. The most potent dose of the TRH mimetic needed to reverse the reserpine-induced hypothermic effect in mice is 0.5 µM/kg, contrasting the 50 µM/kg of TRH needed to obtain the same result. The colonic temperature of mice was registered for 7 h, and areas under the curves for the temperature/time of the subsequent registers were compared between animal groups injected with TRH and tested stereoisomers.

Among the other characteristics of novel TRH stereoisomers are those with low Ki for TRH receptors (as analyzed in rats’ membrane brain preparations; 3.5 vs. 25 nM for TRH); however, when orally administered, enzymatic digestion resistance is crucial to the potency of the analogs. Pharmacokinetics studies have shown that [^3^H]TRH is absorbed in the small intestine but rapidly degraded by enzymes such as thyroliberinase; in contrast, the [^14^C] TRH analog taltirelin (TA-0910) is able to resist degradation and to be absorbed into the blood with its structure intact, which increases its ability to reverse the hypothermic actions of reserpine [36]. Rovatirelin (4S,5S)-5-methyl-N-{(2S)-1-[(2R)-2-methylpyrrolidin-1-yl]-1-oxo-3-(1,3-thiazol-4-yl)propan-2-yl}-2-oxo-1,3-oxazolidine-4-carboxamide 1R-isomer) showed the best anti-hypothermic effect among those tested, and was selected as the candidate for clinical trials [22].

The strategy of synthesizing alternative TRH analogs is helpful for increasing their lipophilicity (versus the lipophilicity of TRH), which could facilitate BBB permeability and accessibility to TRH receptor-expressing areas in the brain, in order to achieve specific actions.

### 1.4. Ischemia/Neuroprotection

Different conditions such as hypoglycemic shock, myocardial infarction, and low blood pressure, and accidents such as drowning may induce ischemic brain episodes, and patients may suffer neuronal death. Particularly susceptible to neuronal loss during those episodes of ischemia are the hippocampal granular cells of the dentate gyrus, which interestingly, exhibit an increased expression of TRH mRNA specifically when starting the reperfusion process after cerebral ischemic injury [25].

This enhanced synthesis of TRH in hippocampal cells after ischemia/reperfusion injury demonstrates that even in unstimulated hippocampal cells from naïve rodents, the expression of the TRH mRNA and the peptide release rise in this area, supporting its central roles in some conditions. Interestingly, this has also been observed in the hippocampus of rats subjected to epileptogenic modelling via electroshocks or in ischemic episodes, in which the peptide has been proposed as a neuroprotector [25,38,39].

After an ischemic episode develops, neuronal death begins; when blood flow is restored, the brain damage may be worsened. Thus, an ischemia/reperfusion injury rodent model has been used to analyze therapeutic factors. The model involves the occlusion of the carotid arteries of rats using the method of electrocauterization, and then a restoration of the blood flow. When TRH proteins and mRNA expression were analyzed in the cells of the dentate gyrus of ischemic/reperfusion injured rats using immunohistochemistry and PCR techniques, respectively, a correlation was found between the enhanced peptide expression and lower rates of hippocampal apoptosis, alongside a better Morris water maze (MWM) performance of injured rats compared with that of sham or saline-injected groups. The transient enhancement of TRH expression (6 to 24 h after injury) in the granular neurons, along with the increased survival of hippocampal cells in the early stages of the ischemic insult, highlights the neuroprotective role of endogenous TRH [25].

These results have prompted the analysis of exogenous administration of peptide as a therapeutic route to improving neuronal survival by delivering TRH to ischemically injured rats. Intracerebroventricular injection of the TRH analog taltirelin hydrate (1 mg/mL) 30 min before (or even 24 h after) inducement of the ischemic episode is able to increase neuroprotection in hippocampal cells by reducing apoptosis. Among the proposed mechanisms of TRH’s actions to increase neuronal survival, the most studied are the elevated turnover of norepinephrine, the antagonizing effect on the opioid system, the activation of the blood flow to brain, the inhibition of cellular death-induced glutamate release, and the stimulation of NMDA receptors [25]. Besides, TRH administration does not have any endocrine effect, since TSH and thyroid hormone serum levels are unchanged.

Findings using injections of TRH and its analogs are not only in agreement with previous observations showing improvements in neuronal survival in rats with cerebral ischemia [39], but importantly, also show that even delayed treatment of ischemia-injured patients using TRH or its analogs may reduce neuronal loss, thereby increasing their recovery odds and supporting the promising therapeutic role of TRH in the clinical setting.

### 1.5. Alzheimer’s Disease/Neuroprotection

The potential role of TRH as a neuroprotector involves using the peptide as a therapeutic agent in psychiatric illnesses such as Alzheimer’s disease. High levels of TRH are observed in the cerebrospinal fluid of AD patients, which support the association of the peptide’s metabolism with the disease.

TRH’s anticonvulsant properties support its role as a neuroprotective agent, and in order to test this effect, in vitro studies have shown that adding TRH to primary cultures of forebrain neurons of fetal rats reduces glutamate-stimulated increases in calcium, which are associated with the development of epileptogenic seizures [40]. TRH’s proposed mechanism of neuroprotection also involves its ability to activate the cholinergic pathway, which ameliorates the cognitive dysfunctions of AD patients and of rodents subjected to AD models. When added to fetal rats’ hippocampal cultured cells, TRH (200 nM, 10 min) stimulates the activity of MAPK and inhibits GSK3β, which prevents the phosphorylation of tau protein [41], a main trigger of the formation of neurofibrillary tangles associated with dementia in AD [26].

### 1.6. Analeptic Effect

Since the 1970s, researchers have tried to demonstrate TRH’s central effects independently of its neuroendocrine role. TRH’s proposed activating role in the CNS of hypophysectomized animals is supported by the direct effect of the peptide (or taltirelin) in shortening the duration of pentobarbital-induced CNS depression in animals [37].

In evaluating TRH’s analeptic actions, concussed mice (by the impact of bakelite rod) that received intramuscular or intravenous injections of TRH tartrate (TRH-T) (0.04–5 mg/kg) in the tail vein showed a decrease in latency for displaying spontaneous movement and right reflexes [27,42]. Additionally, 40% of patients with disturbed consciousness improve their symptoms after 10 days of intramuscular or intravenous administration of TRH-T (0.5–2 mg/kg). The possible neural circuit within which TRH exerts its analeptic function is the cholinergic system, since atropine (a muscarinic antagonist) blocks its activating effects in mice.

Given that previous attempts to evaluate the effectiveness of a TRH-T therapy for protracted disturbances of consciousness are not consistent, recent studies have analyzed the effects of intravenous injections of TRH-T (2 mg/kg) over 2 days in patients with cerebral aneurysm-induced subarachnoid hemorrhage, which may develop neurological alterations [27]. For evaluating the consciousness disturbance and cognitive function of 32 patients, researchers use the score obtained with the Hasegawa dementia rating scale (revised) (HDS-R). When observing the HDS-R score after injecting TRH-T (versus a group of patients not receiving any treatment), the authors observed higher scores in the TRH-T treated group, which supports the efficacy of the analog’s actions in decreasing the effects of dementia. Some 61% of the TRH-T-treated group presented a greater increase in the HDS-R score, which is classified as a good outcome. Some demographic characteristics of patients, such as age (≥60 years) and an initial HDS-R score below 4, represent a risk of obtaining poor responses to TRH-T treatment [27]. These are important aspects to be considered when evaluating the efficacy of TRH-T therapy, because when they are not taken into account, inconsistent outcomes may result.

### 1.7. Spinal Cord Injury (SCI)

Spinal cord injury is a complex pathological process with a prevalence of 20.6 million people and an annual incidence of 0.9 million worldwide [43]; it is characterized by a myriad of cellular and molecular alterations that follow the initial mechanical damage. Nerve tissue damage to the spinal cord may cause different motor alterations and loss of sensibility, depending on the degree of the lesion. The arrival of immune cells to the affected area may cause a scar formed by glia and astrocytes, interrupting correct neuronal communication. There are no fully restorative therapies for SCI [44]; therefore, emerging strategies for its treatment consider the use of trophic factors designed to promote neuronal protection and regeneration [11]. One of these is TRH, which has proven to be effective in improving motor and sensitive functions vs. placebo, at least in patients with incomplete SCI receiving the treatment early after the trauma (dose: 0.2 mg/kg intravenous bolus, followed by 0.2 mg/kg/h infusion over 6 h) [45]. It appears that TRH is able to reduce symptoms caused by SCI, given that it shows anti-inflammatory and antioxidant properties; it functions as an antagonist of leukotrienes and excitotoxins, and increases blood flow in the spinal cord [11].

### 1.8. Spinal Muscular Atrophy (SMA)

SMA is a genetic disorder that causes degeneration of anterior horn cells, which leads to progressive muscle weakness, representing an annual incidence of 1 in 6000 to 1 in 12,000 people. A recent Cochrane review of ten randomized, placebo-controlled trials of treatments for SMA types II and III, with 717 participants, included a double-blind, randomized, placebo-controlled study of nine children undergoing TRH treatment [12]. This study compared the intravenous administration of TRH 0.1 mg/kg once a day in six participants, with three receiving placebo. The duration of the treatment was 29 days, with a follow-up and conclusion of the study at 5 weeks. This small trial does not report any effects of TRH on motor function; additionally, the certainty of evidence of increased muscle strength after 5 weeks of treatment is very low due to the sample size, baseline imbalance, and lack of allocation concealment [46]. The precise mechanism of action of TRH is unknown, but it has been hypothesized that, similarly to its effects on SCI, TRH may have a neurotrophic action on spinal motor neurons [47].

### 1.9. Motor Neuron Diseases

Amyotrophic lateral sclerosis (ALS) and multiple sclerosis (MS) are amongst the most common neurological diseases associated with muscle weakness and physical disability. MS involves upper motor neuron dysfunction, while ALS involves both upper and lower motor neuron dysfunction. While MS is an immune neuroinflammatory condition and ALS is a neurodegenerative disease, both share some symptoms, and may benefit from TRH’s neuroprotective actions.

In the 1980s, there were several clinical trials that focused on evaluating TRH’s role in ALS, since it was known to reduce weakness and spasticity in these patients [48]. Patients with ALS have a low content of TRH in the anterior horn of the spinal cord [49], and TRH therapy may be useful since it influences the excitability of the spinal motor neurons [50].

In humans, the administration of TRH or its analogs via alternate or continuous intravenous infusions, intramuscularly or intrathecally, provoked small-to-moderate improvement in muscle strength and function [51,52,53] due to the peptide functioning as a neuroregulator of the anterior horn cells [54]. In rats, taltirelin has been proven to be a survival factor for developing spinal motor neurons [55], and enhanced neurite outgrowth in a rat embryo ventral spinal cord [56]. Although the autonomic effects were not significant in some studies [57], the undesired side effects depended on the specific features of the disease [58]. Even if these results seemed favorable, some studies found no significant effects after TRH administration [59]; moreover, the transitory, inconsistent, and sex-dependent responses [60,61] overshadowed interest in the peptide as a therapeutic agent for this complex disease. Interestingly, a recently described TRH-based compound (JAK4D), which binds to a pharmacologically different receptor (vs. the one in pituitary) in the human brain, appears to be a promising treatment for different neurodegenerative diseases, and proved to be effective in a mouse model of ALS by attenuating neuronal loss in the lumbar spinal cord [10]. Kelly et al. [10] highlight the importance of studying the therapeutic role of TRH, since it is a neuropeptide with multifaceted actions which overall acts as a “normalizer” of dysregulated physiological systems. The discovery of this novel TRH receptor subtype (which is pharmacologically different from the one already described) will help to draw attention to the use of TRH analogs to treat the symptoms of ALS and other neurodegenerative diseases.

There are fewer studies focused on TRH’s role in the treatment of MS; nevertheless, it is known that TRH expression increases in demyelinating injury sites, as well as in axonal transections (i.e., the main pathological features of MS) [62]. Thus, the authors suggest that TRH may have a protective role during CNS injury responses; though its exact mechanism has not been elucidated, the protection provided against dysmyelination may involve an increase in cerebral blood flow, especially in the brainstem [63].

#### Spinocerebellar Degeneration (SCD)

Dysfunction of the cerebellum results in cerebellar ataxia in patients with spinocerebellar degeneration. TRH-T has been used successfully in Japan to treat cerebellar ataxia since the 1980s [64], and recently, in a mice model of hereditary ataxia, rovatirelin (1, 3, 10 and 30 mg/kg, orally, daily for 2 wk) was shown to be dose-dependently more potent in improving motor function (versus taltirelin). The effect appears to be mediated by an increase in the activity of several brain regions, including the cortex and limbic system, due to TRH receptors mediating any increase in norepinephrine release, and due to an elevation in BDNF expression in the cerebellum, which may decrease neurodegeneration. It is noteworthy that rovatirelin is highly stable in the brain, and its effect on motor function is not attenuated, even after repeated administration [13].

Recently, it has been shown that TRH analog treatment, using an intravenous injection of 2 mg of protirelin tartrate once daily for 14 days in 18 patients with SCD, improved clinically evaluated cerebellar ataxia (using the Scale for the Assessment and Rating of Ataxia [SARA]), which is a measure that reflects improved simple motor execution and lessened dysmetria by evaluating patients’ performance in a prism adaptation task. Given that TRH does not alter the degree of the sensorimotor adaptation process, these two functions seem controlled by different cerebellar mechanisms [14].

Moreover, two randomized double-blind placebo-controlled phase 3 trials in patients with predominant cerebellar symptoms of SCD, show that rovatirelin (1.6–2.4 mg orally, once daily for ~2 yr) promotes a significantly greater reduction in the SARA total score (versus Placebo) from baseline, when both trials are pooled; the effect is more evident in the subgroup of patients with limb and truncal ataxia, and in those with more severe ataxia. Side effects occurred in ≥5% of patients, and included nausea, weight loss and anorexia [15]. The mechanisms of TRH’s actions are increasing various neurotransmitter levels, and providing a neuroprotective effect to improve motor function [65,66].

### 1.10. Fatigue

Fatigue is a common symptom that occurs in many diseases, and after treatments such as chemotherapy. It does not disappear at rest and has negative consequences for quality of life. Mice treated with 5-fluor-uracil (a chemotherapy drug) develop a fatigue-like behavior, as assessed by a decrease in voluntary wheel running and a worse result upon the treadmill fatigue test (TFT). Taltirelin (i.p. at 1 mg/kg, once daily for 6 days, 30 min prior testing) alleviates fatigue-like behavior in the TFT. In a clinical trial, TRH, given as 0.5 mg and 1.5 mg intravenously, caused a change in the visual analog scale of energy from baseline to 7 h after the studied medication infusion [17]. Taltirelin may improve this symptom by modulating several neurotransmitter systems such as orexins, histamine, and GABA, or through TRH’s anti-inflammatory effects [16,17]; moreover, the positive effects of TRH on motor function may be mediated by its receptors in the ventral horn of the spinal cord and can be added to the overall improvement of fatigue symptoms [9]. Since there is no FDA-approved drug treatment for fatigue, taltirelin or another TRH analog may be safe options for people suffering from many diseases, such as cancer [17] or chronic fatigue syndrome.

### 1.11. Food Intake

TRH’s role in energy balance is well established, due to its actions as the main regulator of the hypothalamic–pituitary–thyroid axis, which when activated increased fuel degradation; moreover, TRH has a potent anorectic effect when administered centrally or peripherally in fasted rats (reviewed in [67,68]). Hypothalamic TRH has received more attention as an anorexic agent, given the primordial role of this area in regulating several aspects of energy homeostasis.

Nevertheless, research from our laboratory has also focused on other areas such as the nucleus accumbens (NAc), an important region of the reward neurocircuitry. Pharmacological upregulation of TRH transcription in the hypothalamus decreases feeding by enhancing the hypothalamic peptide levels; in the NAc, high TRH expression causes anxiolysis, which is associated with normal or low ingestive behaviors [69], which is supported by the decrease in food intake and high dopamine release in rats after receiving an injection of TRH directly into the NAc [70]. Moreover, in ad libitum-fed rats, an accumbal TRH injection decreases palatable food intake, but not that of regular chow; interestingly, feeding effects are modulated by stress, and TRH-downregulation facilitates glucocorticoid-induced hyperphagia, which is evident when a glucocorticoid-receptor antagonist (mifepristone) is injected into the NAc; additionally, palatable food intake is reduced while TRH mRNA expression is upregulated [18].

Accumbal TRH’s effect on feeding is mediated by the peptide α-MSH, given that co-administration of α-MSH along with an antisense oligonucleotide directed against pro-TRH mRNA in the NAc impaired α-MSH-induced feeding reductions. Thus, just as in the hypothalamus, TRH mediates some of the effects of α-MSH in hedonic feeding regulation [71]. Given that to our knowledge, there is no recent research in humans regarding the appetite-modulating actions of TRH, this is an area of opportunity in which TRH analogs can be used to decrease food intake in people with a dysregulation in the hedonic drive to eat, such as in patients with obesity or binge-like eating.

### 1.12. Intranasal Delivery of TRH-Nanoparticles

In order to overcome the low BBB permeability of TRH, and to efficiently reach specific brain areas in which it exerts different biological effects, a novel delivery method of the peptide to the brain has been designed and tested in in vivo and in vitro studies. This involves the construction of different nanoparticles (NP) containing the peptide, which may be sprayed into the nasal cavity with an atomizer. Recently, a new hydrophobic polymer (poly sebacic anhydride) (PSA) NP TRH-PSA NP [72,73] has proved to be efficient in loading 100% of TRH and with adequate peptide-releasing properties into the olfactory mucosa, which is similar to the capacity of other previously designed NPs.

Different assays have shown that once in the olfactory neuroepithelium, TRH is easily released from PSA-NPs, and that this process may last up to 12 h, which represents an increase in the peptide’s effective duration; it has also been observed that when used in concentrations of 0.1 µg, TRH-NPs (the dose range used) do not induce toxicity in cultures of A431 human epithelial squamous carcinoma cells [72].

The intranasal delivery of TRH also overcomes its rapid metabolism in stomach, liver, and kidney, as well as its enzymatic degradation in blood by thyroliberinase. This method facilitates an immediate administration of the peptide, which may be needed in some patients, but also makes it unnecessary to increase doses up to amounts that may be toxic and expensive.

When designing an NP, the selection of its formulation is important, since it has to be easily absorbed in the nasal epithelium, thereby overcoming the low lipophilic nature of TRH, which has a pKa ~6.2. This characteristic causes the peptide to be absorbed slowly, subcutaneously, or transdermally, eliminating those routes as a choice of administration for patients who need the drug immediately. The new polymer, PSA-NP, which carries TRH, is permeable in the mucosa and facilitates the peptides’ release into the nasal cavity. When compared to those with PLGA (poly lactic-glycolic acid), PLA (poly-lactic acid) and PCL (poly-caprolactone), it shows similar characteristics for loading TRH and for releasing it intranasally [73].

In vivo, the intranasal application of TRH has been tested to reduce the frequency and intensity of spontaneous seizures in rats subjected to a model of epileptogenesis named electric kindling, supporting the proposed role of TRH as an anticonvulsant [74]. The intranasal delivery of TRH also blocked chemically induced epileptic crisis with pentylenetetrazol (PTZ) injections in rats [74]. This method is promising for increasing the duration of the peptide’s central effects. One of the goals when improving the success of the intranasal delivery of TRH is that of obtaining nanoparticles with a low molecular weight and size that will improve their absorption and their ability to adhere to the mucosa.

In cynomolgus monkeys, the safety of intranasal delivery of PSA NPs carrying TRH has been proved [75], since animals have not shown adverse effects or abnormal behaviors after 28 days of delivery. Further efforts should be made in designing NPs; however, the low toxicity of the ones already tested, their easy biodegradation, cell viability, and their ability to release TRH in the nasal cavity are advantages that make this route of delivery a promising alternative for using TRH in treating many brain alterations.

## 2. Conclusions

**Figure 1:**
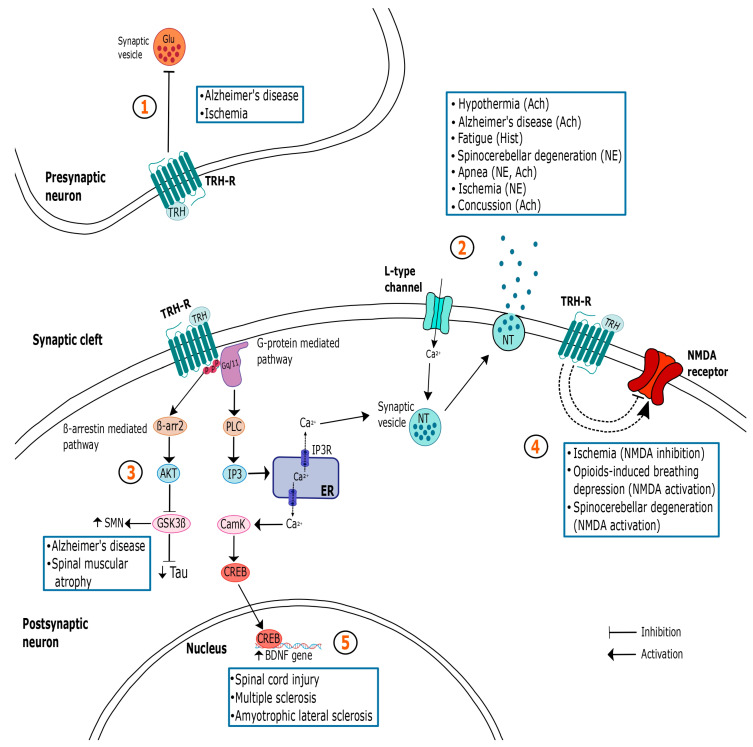
Summary of the different mechanisms of action of TRH in the central nervous system (CNS) and their relationship with the treatment of some pathologies. The myriad of TRH actions in the CNS is paralleled by a complex set of neuronal changes that depend on the region and type of cell analyzed. Importantly, responses are also influenced by the internal state of the animal and are directed by TRH’s role in homeostasis maintenance. It is noteworthy that more than one pathway may be involved in mediating TRH’s actions in the same illness. At the presynaptic neuron, TRH may inhibit glutamate release, acting as a neuroprotective agent and preventing cellular death (1); at the postsynaptic cell, TRH is able to stimulate the release of different NTs (2), thus promoting several effects such as hyperthermia, breathing stimulation, neuroprotection, arousal, analepsia and improved locomotion. TRH’s neuroprotective action may be mediated by GSK3 β inhibition (3), or by the inhibition of NMDA receptors (4). However, when acting as a respiratory activator, it stimulates this receptor. As a neurotrophic factor, TRH may act though BDNF expression (5). In blue squares are the specific conditions that can benefit from TRH administration acting in each pathway. Solid arrows represent well-studied pathways, while dashed lines depict signaling cascades that remain speculative. Ach: acetylcholine; Akt: serine/threonine protein kinase; BDNF: brain-derived neurotrophic factor; β-arr2: β-arrestin 2; CamK: calcium/calmodulin-dependent protein kinase; CREB: cAMP responsive element-binding protein; ER: endoplasmic reticulum; Glu: glutamate; GSK3β: glycogen synthase kinase-3β; Hist: histamine; IP3: inositol 1,4,5-triphosphate; NE: norepinephrine; NMDA: N-Methyl-D-aspartic acid; NT: neurotransmitter; PKC: protein kinase C; SMN: survival motor neuron protein.

TRH is a potential therapeutic drug to be used in treating several neurologic alterations, due to the extensive expression of its type 1 and 2 receptors in different brain areas, with underlying cognitive, motor, arousal, sleep and feeding functions, among others. These brain regions receive dense afferent connections from the TRHergic pathways, wherein the peptide is able to modulate the diverse neurotransmitters’ systems (Figure 1).

Interestingly, TRH acts as a homeostatic factor since it returns specific neural circuits to their basal functioning when perturbed. Given that depending on the state of the animals, the peptide may activate or inhibit the same neurotransmitter pathway, it is considered a state-dependent homeostatic agent. For example, it has been proposed as an antiepileptic as well as an analeptic factor. However, TRH is still not used extensively in the clinical setting due to some disadvantages that are present after its administration. The duration of its effects is short (10 min on average), it has a low permeability to the brain due to its hydrophilicity, it is easily metabolized in the intestines, stomach and in blood, and it may provoke some secondary undesirable effects such as dizziness, nausea, and heart rate alterations.

In this article, we revised the main therapeutic actions of TRH and the new strategies being used to overcome the inconvenient properties of the native peptide, wherein different attempts have been made to demonstrate the improved effects of peptide administration in rodents and humans (in comparison to previous trials). These new approaches include the design and construction of novel TRH analogs with higher potency, a longer half-life, and longer-lasting effects than the peptide and the currently tested mimetics.

Continuous infusions of TRH also attempt to achieve prolonged actions, which are new methods of delivering the peptide (such as the construction of nanoparticles containing TRH that are intranasally administered), thus overcoming its low BBB permeability. The recent efforts described here for using TRH in the clinical setting still present disadvantages or inconsistencies but support the promising potential of the peptide and its analogs as therapeutic agents in a variety of psychiatric and neurological conditions.

## Figures and Tables

**Table 1:** Overview of recent studies regarding the administration of TRH and its analogs for the treatment of different diseases/symptoms.

Disease or Model	Species	Drug and Dose	Main Outcome	Side Effects	Mechanism of Action	Reference
Spinal cord injury	Human	TRH 0.2 mg/kg i.v. bolus followed by 0.2 mg/kg/h infusion over 6 h	Improved motor and sensitive functions	Increased heart rate, flushing, headache, lightheadedness	Neurotrophic antagonist of molecules implicated in tissue injury, increased CNS blood flow	Reviewed in [11]
Spinal muscular atrophy	Human	I.v. TRH 0.1 mg/kg once daily for 29 days	Minor improvement in muscle strength	Abdominal discomfort, flushing, nausea, and vomiting	Neurotrophic effects on spinal motor neurons	Reviewed in [12]
Spinocerebellar degeneration	Mouse	Rovatirelin (1, 3, 10 and 30 mg/kg, orally, daily 2 wk	Improved motor function	NA	Enhanced NE brain activity, decreased neurodegeneration	[13]
	Human	Protirelin (2 mg, i.v. once daily, 14 d)	Improved motor execution and less dysmetria	None	Improved cerebellar long-term depression formation	[14]
		Rovatirelin (1.6–2.4 mg orally, once daily for ~2 yr)	Decreased truncal and limb ataxia	Nausea, weight loss and anorexia	Enhanced neurotransmitter levels, neuroprotective	[15]
Fatigue	Mouse	Taltirelin (i.p. at 1 mg/kg, once daily, 6 d)	Decreased fatigue-like behavior	NA	Enhanced neuromodulation of histamine, orexins, GABA, anti-inflammatory	[16]
	Human	TRH (0.5 mg and 1.5 mg i.v. once a wk for 4 wk	Improvement in energy levels	Increased blood pressure, heart rate, nausea, flushing		[17]
Stress-induced hyperphagia	Rat	TRH intra-NAc (3 μg/0.5 μL)	Decreased palatable food intake	NA	Modulation of glucocorticoid receptor signaling	[18]
Opioids-induced respiratory depression	Anesthetized rats	TRH Intra-tracheal (IT) (5 mg/kg) Taltirelin IT (2 mg/kg) TRH i.v. (1 mg/kg plus 5 mg/kg/h) Taltrelin I.v. (1 mg/kg)	Increased breathing rate, reversed morphine’s effects on ventilation	Taltirelin causes elevation in blood lactic acid; TRH does not normalize arterial carbon dioxide and oxygen levels	Intracellular signaling of the activated TRH-receptor may bias the receptor downstream from the mu opioid receptor	[19]
	agotomized artificially ventilated rats	TRH (100 nM) i.v. or into the dorsal medulla	Antagonize morphine-induced respiratory depression, as measured by diaphragmatic activity	NA	By acting at N-methyl-D-aspartate acid (NMDA) receptors	Reviewed in [20]
	In vitro brainstem-spinal cord preparation from 1- to 4-day-old rats	TRH (100 nM)	Antagonized morphine respiratory depression	NA	NA	Reviewed in [20]
	Humans	TRH i.v. repeated infusions of TRH (0.2–8 mg) There are no studies with taltirelin in humans	No effect	NA	Poor penetration of TRH into the brain compartment	Reviewed in [21]
Hypothermia	Mice	TRH i.v. (50 μmol/Kg). Rovatirelin and other analogs i.v. (0.50 μmol/Kg)	Antagonizes reserpine-induced hypothermia	NA	TRH and its analogs increase catecholamines’ secretion and stimulate the sympathetic nervous system	[22]
	Mice	TRH, oral (50 µmol/Kg/mL) Different analogs oral, (5 and 10 µmol/Kg/mL)	Antagonizes reserpine-induced hypothermia	NA	TRH and its analogs increase catecholamines’ secretion	[23]
Obstructive sleep apnea	Rats	Taltirelin, i.p. (0.1 and 1 mg/kg)	Stimulates pharyngeal muscle and tongue motor activities	Induces wakefulness	High expression of TRH receptors in the hypoglossal motor nucleus	[24]
Ischemia/neuroprotection	Rats	Taltirelin, i.c.v. (1 mg/mL)	Reduces apoptosis in the hippocampus	NA	Increases turnover of NE, inhibits glutamate release	[25]
Alzheimer’s disease/neuroprotection	Humans, cultures of rat fetal hippocampal cells	TRH i.v. 0.1 mg/kg to humans TRH 200 nM, 10 min to cultured rats’ hippocampal cells.	Ameliorates cognitive dysfunctions in AD patients and in rodent models of the illness	Transiently raises systolic blood pressure	Activates cholinergic system and reduces Tau phosphorylation in cultured rat hippocampal neurons	Reviewed in [26]
Unconsciousness/concussion	Humans	TRH-tartrate, i.v. (2 mg)	Improved scores in Hasegawa dementia rating scale (revised)	NA	Activation of the cholinergic system	[27]

i.c.v.: intracerebroventricular; i.p.: intraperitoneal; i.v.: intravenous; NE: norepinephrine; NA: not assessed.

## Data Availability

Not applicable.
